# Towards the Development of an Integrative, Evidence-Based Suite of Indicators for the Prediction of Outcome Following Mild Traumatic Brain Injury: Results from a Pilot Study

**DOI:** 10.3390/brainsci10010023

**Published:** 2020-01-02

**Authors:** Aleksandra Gozt, Melissa Licari, Alison Halstrom, Hannah Milbourn, Stephen Lydiard, Anna Black, Glenn Arendts, Stephen Macdonald, Swithin Song, Ellen MacDonald, Philip Vlaskovsky, Sally Burrows, Michael Bynevelt, Carmela Pestell, Daniel Fatovich, Melinda Fitzgerald

**Affiliations:** 1 Curtin Health Innovation Research Institute, Curtin University, Bentley, WA 6102, Australia; a.gozt@postgrad.curtin.edu.au (A.G.); ab2458@bath.ac.uk (A.B.); carmela.pestell@uwa.edu.au (C.P.); 2 Perron Institute for Neurological and Translational Science, Nedlands, WA 6009, Australia; 3 Telethon Kids Institute, West Perth, WA 6005, Australia; Melissa.Licari@telethonkids.org.au; 4 School of Biological Sciences, The University of Western Australia, Crawley, WA 6009, Australia; allison.halstrom@uwa.edu.au (A.H.); hm525@bath.ac.uk (H.M.); stephen.lydiard@bath.edu (S.L.); 5 Emergency Medicine, The University of Western Australia, Crawley, WA 6009, Australia; glenn.arendts@uwa.edu.au (G.A.); Stephen.Macdonald@health.wa.gov.au (S.M.); Daniel.Fatovich@health.wa.gov.au (D.F.); 6 Centre for Clinical Research in Emergency Medicine, Harry Perkins Institute of Medical Research, Nedlands, WA 6000, Australia; Ellen.MacDonald@health.wa.gov.au; 7 Emergency Department, Royal Perth Hospital, Perth, WA 6000, Australia; 8 Radiology Department, Royal Perth Hospital, Perth, WA 6000, Australia; Swithin.Song@health.wa.gov.au; 9 School of Medicine, The University of Western Australia, Crawley, WA 6009, Australia; philip.vlaskovsky@uwa.edu.au (P.V.); sally.burrows@uwa.edu.au (S.B.); 10 School of Surgery, The University of Western Australia, Crawley, WA 6009, Australia; Michael.Bynevelt@health.wa.gov.au; 11 Neurological Intervention and Imaging Service of Western Australia, Sir Charles Gardener Hospital, Nedlands, WA 6009, Australia; 12 School of Psychological Science, The University of Western Australia, Crawley, WA 6009, Australia

**Keywords:** persistent post-concussion symptoms, blood-based biomarkers, neuropsychological assessment, MRI, prediction

## Abstract

Background: Persisting post-concussion symptoms (PPCS) is a complex, multifaceted condition in which individuals continue to experience the symptoms of mild traumatic brain injury (mTBI; concussion) beyond the timeframe that it typically takes to recover. Currently, there is no way of knowing which individuals may develop this condition. Method: Patients presenting to a hospital emergency department (ED) within 48 h of sustaining a mTBI underwent neuropsychological assessment and demographic, injury-related information and blood samples were collected. Concentrations of blood-based biomarkers neuron specific enolase, neurofilament protein-light, and glial fibrillary acidic protein were assessed, and a subset of patients also underwent diffusion tensor–magnetic resonance imaging; both relative to healthy controls. Individuals were classified as having PPCS if they reported a score of 25 or higher on the Rivermead Postconcussion Symptoms Questionnaire at ~28 days post-injury. Univariate exact logistic regression was performed to identify measures that may be predictive of PPCS. Neuroimaging data were examined for differences in fractional anisotropy (FA) and mean diffusivity in regions of interest. Results: Of *n* = 36 individuals, three (8.33%) were classified as having PPCS. Increased performance on the Repeatable Battery for the Assessment of Neuropsychological Status Update Total Score (OR = 0.81, 95% CI: 0.61–0.95, *p* = 0.004), Immediate Memory (OR = 0.79, 95% CI: 0.56–0.94, *p* = 0.001), and Attention (OR = 0.86, 95% CI: 0.71–0.97, *p* = 0.007) indices, as well as faster completion of the Trails Making Test B (OR = 1.06, 95% CI: 1.00–1.12, *p* = 0.032) at ED presentation were associated with a statistically significant decreased odds of an individual being classified as having PPCS. There was no significant association between blood-based biomarkers and PPCS in this small sample, although glial fibrillary acidic protein (GFAP) was significantly increased in individuals with mTBI relative to healthy controls. Furthermore, relative to healthy age and sex-matched controls (*n* = 8), individuals with mTBI (*n* = 14) had higher levels of FA within the left inferior frontal occipital fasciculus (*t* (18.06) = −3.01, *p* = 0.008). Conclusion: Performance on neuropsychological measures may be useful for predicting PPCS, but further investigation is required to elucidate the utility of this and other potential predictors.

## 1. Introduction

Mild traumatic brain injury (mTBI), also known as concussion, accounts for approximately 70–80% of all traumatic brain injuries worldwide [1]. mTBIs are caused by the head hitting an object or by forceful mechanical impacts external to the body that result in an abrupt acceleration/deceleration of the craniocervical complex [2]. mTBIs are characterised by a rapid onset of transient changes in neurological function which manifest as physical, cognitive, psychological/emotional, and sleep-related signs and symptoms [3]. Whilst most patients who sustain a mTBI present with their own unique constellation of symptoms [4], the most commonly reported symptoms include headache, difficulties in concentrating and maintaining attention, and alterations in mood and sleep [5]. Symptoms typically resolve within two weeks following injury [6,7,8], however, 10–20% of individuals who have sustained a mTBI continue to experience mTBI-related symptoms for months to years [9,10]. These individuals are said to be suffering from a complex condition known as persisting post-concussion symptoms (PPCS) [10,11,12]. PPCS is associated with significant disability [13,14,15,16,17] and a heightened use of health services [18,19], making it an emergent public health issue.

Previous studies have identified factors that can predict PPCS, however, these have not been found to be sufficiently precise to use on an individual patient basis. Having the ability to identify individuals at-risk of developing PPCS is necessary for both clinical and research practice. For clinicians, prognostic models would assist in tailoring treatment plans to better suit the needs of the individual and, more importantly, facilitate the early provision of targeted treatment strategies to circumvent ongoing problems [20]. Moreover, researchers could use prediction models to help enrich clinical trials in order to accelerate the development of evidence-based therapies [21] that aim to prevent or ameliorate the effects of PPCS, as well as other neurodegenerative diseases that have been found to be associated with mTBI, such as Alzheimer’s disease [22,23,24] and chronic traumatic encephalopathy [25,26,27,28,29,30].

A range of demographic and injury-related factors, as well as neuropsychological, physiological and structural measures have been investigated for their ability to predict outcome following mTBI. However, variations in study methodologies have often resulted in conflicting results being reported [31], and many of the studies conducted thus far have been limited to investigating only one or a small subset of prognostic factors [32]. Demographic and injury-related factors have received considerable attention, partly because of the convenience with which they can be extracted from medical records. Of those examined, factors frequently associated with increased incidence of PPCS include being female [33,34,35,36], previous history of mTBI [16,37], and affective and anxiety-related psychological disorders [16,38]. A variety of neuropsychological measures have also been examined as possible predictors of PPCS because cognitive deficits have been observed in both individuals who have sustained a mTBI [39,40] and those suffering from PPCS [41]. In particular, individuals who perform poorly on tasks of executive function [42], memory [33,43,44,45], and psychomotor function [21] have been found to be at a heightened risk of developing PPCS. However, the fidelity with which neuropsychological measures can prognosticate PPCS has been questioned, as individual performance can be confounded by extraneous factors such as age, socio-economic status, and prior education [46,47,48,49]. Hence, there is a need to identify and examine the prognostic capabilities of additional objective physiological and morphological variables.

Blood-based biomarkers are surrogate markers of disease that can be quantified from blood samples, and present as an option for use as diagnostic and prognostic indicators as they are a relatively cost-effective means of assessing the physiological mechanisms underpinning the condition of interest [50]. Of particular relevance to mTBI are blood-based biomarkers pertaining to neuronal injury, such as neuron-specific enolase (NSE) and neurofibrillary protein-light (NFL), as well as glial structure and function, such as glial fibrillary acidic protein (GFAP). These biomarkers are constrained to their respective locations in the cellular environment under normal physiological conditions, however following events where cellular membrane integrity is compromised, these biomarkers are released into the extracellular space [51,52,53,54,55,56,57,58,59,60]. In mTBI, damage to the cellular membrane primarily occurs as a result of mechanical forces that are present at time of injury [61]. Although there is a lack of normative values associated with any of these biomarkers, a relatively elevated presence of NSE, NFL, and GFAP in blood samples is considered to be indicative of neuronal injury, axonal injury, and astrocytic damage and possible blood–brain barrier disruption [62,63].

Diffuse axonal injury (DAI) is also thought to play a role in maintaining mTBI symptoms [64,65], through disruptions to axolemma and neurofilament organisation that result in compromised structural integrity of white brain matter [66]. DAI can be identified and quantified through the use of diffusion tensor imaging (DTI), an advanced magnetic resonance imaging technique that estimates the displacement of water molecules in biological tissue [67,68]. A growing body of literature suggests that certain DTI parameters may serve as biomarkers for the microstructural damage to white brain matter seen in mTBI [66], including fractional anisotropy (FA) and mean diffusivity (MD) [65,69]. FA is a normalised measure that describes the degree to which diffusion of water is unidirectional in each voxel [70], and is an indicator of complex biophysiological processes such as axonal density [64,69,71,72,73]. MD corresponds to the average apparent diffusion coefficient measured along the three principal diffusion directions in a voxel [65]. Disease processes that affect the integrity of the cellular membrane are known to affect MD [74,75], which is considered to be a non-specific, albeit sensitive, measure of alterations to brain tissue [76]. Although several studies have reported differences in DTI scalars amongst mTBI patients, research into whether they could be used to predict PPCS is limited.

Given that PPCS is a complex multifactorial condition that affects several aspects of functioning, it is unlikely that any single factor will be sufficiently capable of predicting the disorder at the individual level. Moreover, the predictive power of models is found to increase when several measures of clinical assessment (e.g., symptom, neurocognitive, balance) are considered together [21]. This suggests that model accuracy is likely to be optimised when a multidimensional approach that acknowledges the neurobiopsychosocial aspects of PPCS is undertaken. Thus, the aims of the present pilot study were twofold. Firstly, to evaluate the potential for a suite of demographic, neuropsychological, blood-based biomarker and MRI-DTI outcomes to be incorporated into future multivariate analyses aiming to predict PPCS. Secondly, to contribute data for the blood-based biomarker and MRI outcomes for which there is currently relatively little literature regarding the differences between mTBI and controls. These two aims were addressed in this small pilot study with the intention that the outcomes be validated in a larger scale study designed to generate a suite of outcomes that could be used to predict PPCS.

## 2. Methods

### 2.1. Participant Recruitment and Inclusion Criteria

Recruitment for this prospective, observational pilot study took place between September 2015 and January 2018. Participants were patients presenting to the Emergency Department at Royal Perth Hospital (RPH), Western Australia, with mTBI. mTBI was defined in accordance to criteria specified by the American Association of Neurological Surgeons (https://www.aans.org/Patients/Neurosurgical-Conditions-and-Treatments/Concussion). That is, patients verbally verified that their closed head injury resulted from mechanical force or trauma and involved an immediate and transient alteration in brain function, including alteration of mental status and level of consciousness. Participants were enrolled into the study if they were aged between 18 and 50 years of age, presented to the ED within 48 h of head injury with symptoms that were attributable to that injury, and cranial CT scan revealed no presence of intracranial injury or CT was not performed. Participants were excluded if at time of presentation to the ED they scored 13 or less on the Glasgow Coma Scale (GCS), were a ward of state, prisoner, or under a mental health treatment order, were unsuitable for undertaking MRI procedures according to standard practice (e.g., metal implants), were homeless, were substance dependent, their head injury was deemed to be entirely due to primary seizure, were non-English speaking; or reported to suffer from pre-existing cognitive impairment. All MRI scans performed were reviewed by a consultant neuroradiologist, and any participants identified with abnormality not associated with mTBI were excluded from the study. A cohort of age and sex-matched, uninjured controls were recruited from the community to serve as a comparison group for neuropsychological and blood-based biomarker outcomes, while a separate cohort of healthy, age and sex-matched uninjured community-dwelling participants was recruited as MRI control subjects. Ethics approval for the study was obtained through the RPH Human Ethics Committee (RPH Ethics Approval Number REG 15-062/ANZCTR:123615000543583).

### 2.2. General Data Collection Protocol

Study participants were recruited by on-duty research nursing staff during daylight hours. Written consent was obtained directly from participants, or from their accompanying next-of-kin and then reconfirmed by the participants. Following consent, blood samples were procured by nursing staff and trained research assistants administered the neuropsychological testing battery, both of which occurred within 48 h of mTBI injury. A convenience subset of participants was later referred to the Radiology Department at RPH for MRI.

At ED presentation, participants were invited to attend a follow-up assessment that was scheduled approximately 28 days later. This involved the procurement of another non-fasting blood sample and re-administration of the neuropsychological test battery. Alternative forms of the neuropsychological tests where used where possible (i.e., RBANS^®^ Update Test form B).

### 2.3. Measures

#### 2.3.1. Neuropsychological Test Battery

A custom neuropsychological test battery was compiled to assess the physical, cognitive, and psychological symptoms associated with mTBI. More specifically, the physical symptoms associated with mTBI were measured using the Rivermead Post-Concussion Symptoms Questionnaire (RMPCQ) [77]. This self-report measure, which has been used to evaluate PPCS in previous prognostic studies [37,42,43,44,45,78,79,80,81,82], comprises of 16 symptoms that are commonly experienced following head injury. Respondents indicate the severity of their symptoms over the past 24 h (h) on a five-point Likert scale (0 = ‘none’, 4 = ‘severe’), relative to their experience before sustaining their mTBI. Total scores range from 0 to 64, with higher scores indicating increased severity of symptoms being experienced by participants.

Cognitive functioning, namely the domains of attention, language and memory was measured by the Repeatable Battery for the Assessment of Neuropsychological Status Update (RBANS^®^ Update) [83]. The RBANS^®^ Update comprises of five subscales: Immediate Memory, Visuospatial/Constructional Ability, Language, Attention and Delayed Memory, which like the original RBANS^®^ are combined to produce a composite Total Score [84]. Executive function was also measured using the Trails Making Test B (TMT B) [85]. Briefly, this measure comprises an array of numbers and letters that are individually presented within circles. To complete the task, examinees are instructed to connect all items in the correct numerical and alphabetical orders, whilst switching between the two sets as quickly and accurately as possible. Examinees are timed and are allowed a maximum of 5 min to complete the task. Longer completion times indicate slower, or impaired, executive functioning capabilities.

Mood was measured using the Depression, Anxiety and Stress Scales-21 item version (DASS-21) [86]. The DASS-21 is a self-report questionnaire consisting of 21 items which has been designed to provide a quantitative measure of an individual’s subjective experience of three related emotional states (subscales): depression, anxiety and stress. Individuals complete the DASS-21 by indicating the frequency with which they have experienced the listed symptoms on a four-point Likert Scale (i.e., 0 = items did not apply to me at all: ‘never’ to 3 = Item applied to me very much, or most of the time: ‘almost always’) over the week prior to mTBI. Individual’s scores are summed and interpreted according to provided guidelines for each subscale, with higher scores indicating greater severity of symptoms experienced by the individual.

Lastly, effort was evaluated using the Rey 15-item Memory Test [87], a brief visual memory test that is frequently used as a screening measure to assess symptom validity and/or feigned memory impairment [88]. The test involves presenting individuals with a 3 × 5 matrix of meaningful symbols for a duration of 10 s before asking them to freely recall or draw the items that they can remember in the correct sequence. Individuals unable to recall at least 9 of the 15 items (that is, at least 3 of the 5 character sets) can be suspected of malingering [85] (p. 778). Overall, the total time taken to complete the neuropsychological tests spanned approximately 40 min on both testing occasions.

Consistent with the study’s ethics approval, each individual participants’ neuropsychological test results were reviewed by research assistants. Cases of concern (e.g., elevated scores on the RMPCQ, DASS-21 scores and/or impaired performance on tests of cognitive and executive function) were referred to the chief neuropsychologist (C.P.), and in cases where physical symptomatology was prevalent; to the emergency physician (D.F.), for further clinical review. Moreover, psychological counselling was offered free-of-charge to all study participants for any distress or symptomatologies that they may have experienced relating to their mTBI incident. This was coordinated by C.P. at the Robin Winkler Clinic at the University of Western Australia.

#### 2.3.2. Blood Collection and Blood-based Biomarker Quantification

Non-fasting blood samples were collected from patients by on-duty research nursing staff at presentation to the ED and stored at −80 °C until use. Biomarker concentrations in plasma (GFAP) and serum samples (NFL, NSE) were measured with a digital array technology (Quanterix Corporation; Lexington, Massachusetts: USA) that uses a highly sensitive single molecule enzyme-linked immunoarray (Simoa™) method previously described [89]. More specifically, levels of GFAP were determined using Simoa™ GFAP Discovery Kit (Product number 102336, Lot 501277), while NFL and NSE were determined using the Simoa™ NF-Light Advantage Kit (Product Number 103186, Lot 501213) and NSE Discovery Kit (Product Number 102475, Lot 501345), respectively. Blood-based biomarkers were quantified on two separate occasions in two batches to minimise duration of storage prior to analysis (June 2017 and August 2018). Concentrations of blood-based biomarkers were multiplied by the dilution factor to generate corrected concentrations.

#### 2.3.3. MRI Data Collection

Imaging was conducted at the Department of Radiology at Royal Perth Hospital using a 3T Siemens Skyra scanner (Siemens Healthcare, Erlangen, Germany) and a 32-channel head coil. Sequences included an axial 3D T1-weighted gradient echo sequence (TE/TI/TR = 2.48/900/2200 ms, flip angle = 8°, field of view (FOV) = 230 × 230 mm^2^, matrix size = 256 × 256, slice thickness = 1 mm, voxel size = 1 × 1 × 1 mm^3^) and DTI using a spin echo-planar sequence (TE/TR = 110/8800 ms, b = 3000 s/mm^2^, 64 directions, one average, FOV = 240 × 240 mm^2^, matrix size = 96 × 96, 60 slices, slice thickness = 2.5 mm, voxel size = 2.5 × 2.5 × 2.5 mm^3^). Total scan time took approximately 40 min.

### 2.4. Diagnosis of PPCS

Participants were classified as experiencing PPCS if at the 28-day follow-up they scored in the moderate (25–32 points) to severe (33+ points) range on the *RMPCQ* [90]. Individuals who did not meet this criterion were considered to have experienced typical mTBI recovery.

### 2.5. Statistical Analyses

Data are summarised using means and standard deviations (SD) or counts and proportions, as appropriate. Data were screened for extreme outliers defined according to Tukey’s outlier detection method; that is, equaling to or exceeding 3 times the inter-quartile range (3xIQR) below the first quartile or above the third quartile. Outliers meeting this criterion were investigated and determined to be implausible values and removed prior to analyses: only 2 GFAP values and 1 NSE value met these criteria. All statistical analyses were conducted using IBM SPSS software v25 (Armonk, NY, USA), except for univariate exact logistical regressions which were performed using SAS software v9.4 (Cary, NC, USA), and *p* < 0.05 was used to indicate statistical significance, unless otherwise specified. No adjustment for multiple testing was made due to the exploratory nature of the study.

#### Predictors of PPCS

Univariate exact logistic regression was conducted in order to determine the odds of an individual developing PPCS based on the demographic, injury characteristic, neuropsychological, and blood-based biomarker data collected. Results are reported as odds ratios (OR) with 95% confidence intervals (95% CI).

Conditional logistic regression was conducted to determine whether blood-based biomarkers examined could discern between individuals who had sustained a mTBI, regardless of recovery status, and healthy controls. In this instance, individuals with mTBI that were identified as an outlier, or for whom blood samples were not available, were excluded from the analyses, along with their matched healthy controls.

### 2.6. MRI Data Analyses

Due to the small number of individuals who sustained a mTBI, underwent MRI and were classified as having PPCS, all neuroimaging data collected was examined for between-group differences amongst individuals who sustained a mTBI, regardless of their recovery status, and healthy controls.

#### 2.6.1. Tract-Based Spatial Statistics

DTI processing and voxelwise statistical analysis were carried out using FMRIB Software Library (FSL; http://www.fmrib.ox.ac.uk/fsl, [91], using tract-based spatial statistics [92]). First, FA images were created by fitting a tensor model to the raw diffusion data using FMRIB’s Diffusion Toolbox (FDT), and then brain-extracted using the Brain Extraction Tool [93]. All participants’ FA data were then aligned into a common space using the FSL nonlinear registration tool (FNIRT) [94,95], which uses b-spline representation of the registration warp field [96]. Next, the mean FA image was created and thinned to create a mean FA skeleton which represents the centres of all tracts common to the group. Each subject’s aligned FA data was then projected onto this skeleton and the resulting data fed into voxelwise cross-subject statistics. Clusters were tested for significance at *p* ≤ 0.05, corrected for multiple comparisons across space using the Threshold-Free Cluster Enhancement (TFCE) approach.

#### 2.6.2. Region of Interest Analyses

FA and MD values were computed for a selection of regions of interest (ROIs), which were chosen based on previous findings reported in the literature indicating that they were either affected following mTBI or implicated in the neuropsychological functions that were assessed by the measures used in the present study. More specifically, the ROIs examined were the anterior corona radiata [97], the anterior, retrolenticular, and posterior components of the internal capsule [64,97,98], cingulum [64,99], corpus callosum [67,97,100], the superior and inferior fronto-occipital fasciculi [67] as well as the superior and inferior longitudinal fasciculi [64,67,101]. All ROIs were evaluated in both left and right hemispheres apart from the corpus callosum, which was examined as a whole as well as genu, body, and splenium segments.

Maps of the ROIs were created first, using the atlas panel featured in FSLeyes (v2.1). All ROIs were located on either the JHU ICBM-DTI-81 White-Matter Labels or the JHU White-Matter Tractography Atlases. All ROI masks were then binarized using FSLmaths, with a threshold of 30 being applied to masks for ROIs that were identified using a probabilistic map (i.e., JHU White-Matter Tractography Atlas). FA and MD values were then extracted for each participant for all ROIs using ‘fslmeants’.

Extracted FA and MD values were then exported to IBM SPSS software to evaluate between-group differences. Data corresponding to each ROI was screened for outliers as per Tukey’s outlier detection method described above. *t*-tests were conducted in order to examine whether there were between group differences in FA and MD of the ROIs extracted. Partial correlations controlling for the effects of age, sex and time elapsed between presentation at the ED and MRI scanning procedures, were subsequently conducted to examine whether there was a correlation between extracted ROI FA and MD values and participants’ performance on outcome measures pertaining to neuropsychological functions previously reported in the literature.

## 3. Results

### 3.1. Study Sample

**Figure 1:**
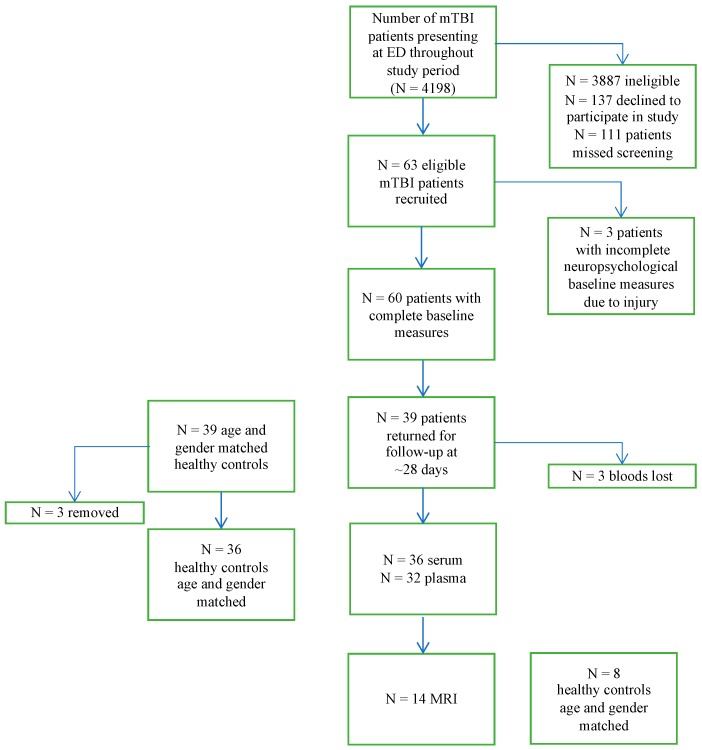
Consort diagram of study sample.

A total of *n* = 63 participants who had sustained a mTBI were enrolled into the study. Of these, *n* = 60 had a complete data set for measures obtained at presentation to the ED, *n* = 39 presented at follow-up ~28 days later and of these, *n* = 36 completed all elements of neuropsychological testing. Details of eligibility and missing data are provided in Figure 1. On average, patients in the *n* = 36 study sample presented to the ED 10.86 h following injury (SD = 10.49, Range = 1.25–45 h, *n* = 35) and attended follow up 34.61 days later (SD = 7.19, Range = 25–55 days); MRI scanning procedures were conducted on average 30.07 days following injury (SD = 18.78; Range = 3–60 days). No participants required intervention by the neuropsychologist, emergency physician or neuroradiologist, or requested the use of these services.

### 3.2. Characteristics of Participants Included in the Study and Participants Lost to Follow-Up

Descriptive statistics for individuals who did and did not return for follow-up in this study are presented in Table 1. Unfortunately, a number of logistical difficulties were experienced during the establishment of processes early in the study and these partially account for the relatively low retention rate of participants in the present study. Statistical tests have been performed to assess differences between groups, however, results need to be interpreted in light of the present study’s small sample size and resulting low power and thus, non-statistically significant results do not necessarily indicate no difference between the groups. As such, the data appear to suggest that a history of headache/migraine and neurological disorder was more prominent amongst individuals who presented for follow-up, relative to those individuals who did not present for follow-up. Furthermore, a lower proportion of individuals who presented for follow-up reported a history of mTBI and psychological disorder relative to those individuals who did not present for follow-up. In addition to this, participants who presented for follow-up also tended to score higher on the RBANS^®^ Update Total, Immediate Memory, and Attention Index scores, and were faster at completing the TMT B at presentation to the ED, relative to those who did not.

### 3.3. Characteristics of Patients with mTBI and PPCS

**Figure 2:**
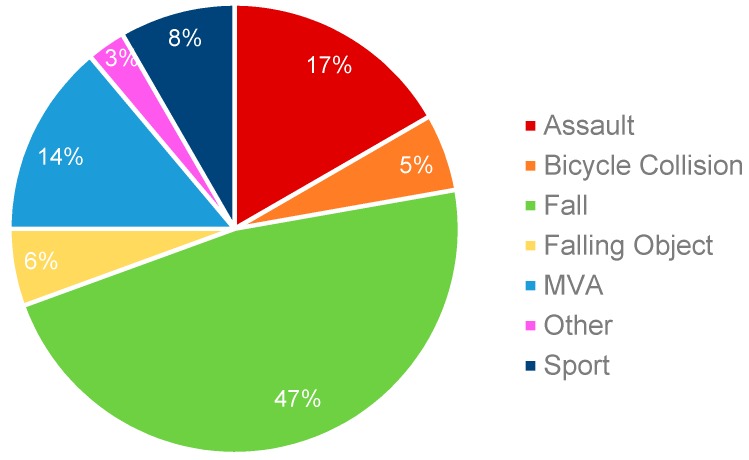
Breakdown of mTBI by type of causal mechanism (*n* = 36).

The cause of mTBI varied amongst study participants, with the most frequent cause being falls (47%) (Figure 2).

Of the individuals that returned for follow-up, *n* = 3 (8.33%) were classified as having PPCS. For patients classified as having PPCS, the causal mechanisms of mTBI were falls (*n* = 2) and falling objects (*n* = 1). Descriptive statistics suggest that individuals in the study sample who were diagnosed with PPCS may have been younger relative to those that recovered typically from mTBI, and all reported a history of prior mTBI. There were no statistically significant differences across the other demographic and injury-related characteristics surveyed (Table 2).

### 3.4. Predictors of PPCS

**Figure 3:**
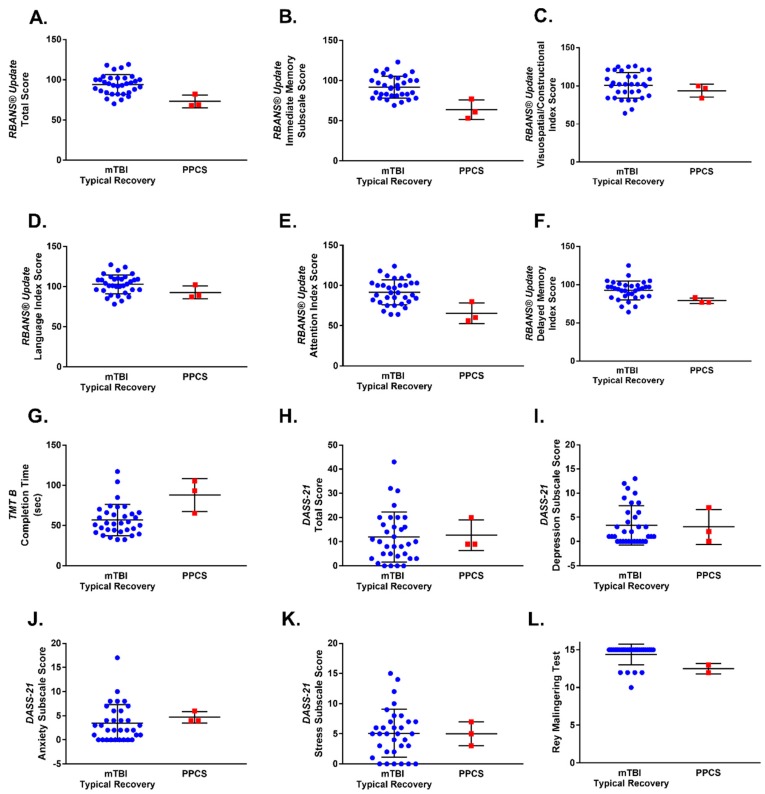
Scatterplots depicting patient performance on neuropsychological outcomes, as measured at presentation to the ED. Mean and standard deviation are shown for mTBI typical recovery and PPCS groups for all neuropsychological outcomes assessed, which were as follows: (**A**) the RBANS^®^ Update Total Score, (**B**) RBANS^®^ Update Immediate Memory subscale, (**C**) RBANS^®^ Update Visual/Constructional subscale, (**D**) RBANS^®^ Update Language subscale, (**E**) RBANS^®^ Update Attention subscale, (**F**) RBANS^®^ Update Delayed Memory subscale, (**G**) TMT B, (**H**) DASS-21 Total Score, (**I**) DASS-21 Depression subscale, (**J**) DASS-21 Anxiety subscale, (**K**) DASS-21 Stress subscale, (**L**) Rey Malingering Test.

None of the demographic variables or injury-related characteristics surveyed were found to be statistically significant predictors of PPCS in this pilot study (Table 2). Amongst individuals who had sustained a mTBI, increased performance on the RBANS^®^ Update Total Score (OR = 0.81, 95% CI: 0.61–0.95, *p* = 0.004), and the RBANS^®^ Update Immediate Memory (OR = 0.79, 95% CI: 0.55–0.94, *p* = 0.001) and Attention (OR = 0.86, 95% CI: 0.71–0.97, *p* = 0.007) indices at presentation to the ED suggested a decreased odds of developing PPCS. Faster completion of the TMT B at ED presentation also suggested decreased odds of developing PPCS (OR = 1.06, 95% CI: 1.00–1.12, *p* = 0.032). No significant associations were detected between PPCS and performance on the other neuropsychological measures examined (Table 2). Scatterplots illustrating individual mTBI and PPCS patient performance on each of the neuropsychological outcomes as measured at ED presentation are depicted in Figure 3.

**Figure 4:**
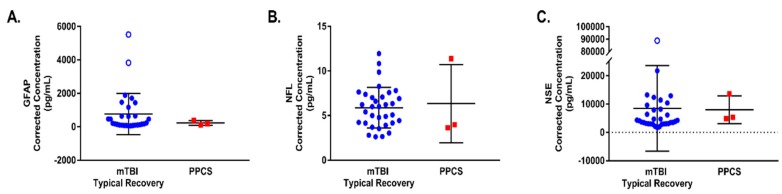
Scatterplots depicting the corrected concentrations of blood-based biomarkers (**A**) GFAP, (**B**) NFL and (**C**) NSE measured from blood samples obtained upon patient presentation to the ED. Mean and standard deviation are shown for mTBI typical recovery and PPCS groups for all outcomes assessed. Outliers are denoted by empty circles (mTBI Typical Recovery) and squares (PPCS), which were excluded from OR analyses presented in Table 3.

None of the blood-based biomarkers examined were found to be statistically significant predictors of PPCS in this pilot study (Table 3). As an example, for GFAP the OR of 0.998 for a 1 pg/mL change equates to an OR of 0.905 for a 50 pg/mL change. The corrected concentrations of blood-based biomarkers measured in blood samples from individual mTBI and PPCS patients, collected at presentation to the ED, are presented as scatterplots in Figure 4.

### 3.5. Differences in Biomarkers between mTBI and Healthy Controls

**Figure 5:**
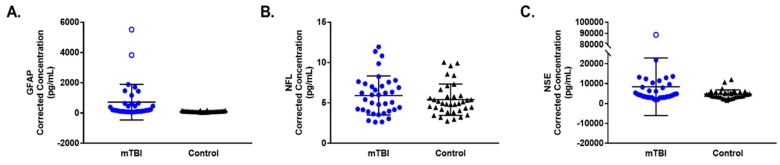
Scatterplots depicting the corrected concentrations of blood-based biomarkers (**A**) GFAP, (**B**) NFL and (**C**) NSE measured from blood samples obtained upon patient presentation to the ED. Mean and standard deviation are shown for individuals who sustained a mTBI (mTBI) and healthy controls (control) for all biomarkers assessed. Outliers are denoted by empty circles (mTBI), which were excluded from OR analyses presented in Table 4.

A significant association was identified between GFAP and mTBI where a one unit increase in GFAP generated a 2.8% increase in the odds of mTBI compared to healthy controls (OR = 1.028, 95% CI: 1.001–1.056, *p* = 0.042). Note that the OR of 1.028 for a 1 pg/mL change equates to an OR of 3.978 for a 50 pg/mL change. No significant association was detected between the odds of mTBI and levels of NSE or NFL (Table 4). However, the estimated effect size for NFL warrants further investigation. Blood-based biomarker concentrations from mTBI patients obtained at ED presentation (regardless of recovery status) and from healthy control participants are presented as scatterplots in Figure 5.

### 3.6. Neuroimaging Outcomes

**Figure 6:**
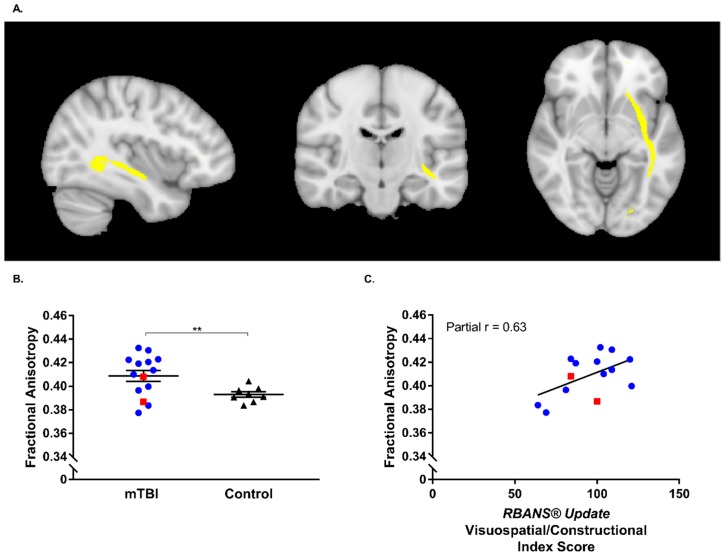
(**A**) Left inferior fronto-occipital fasciculus (IFOF) threshold of 30 presented in radiological view (voxel coordinates 128,108,65); (**B**) Scatterplot depicting fractional anisotropy values extracted from the left IFOF threshold of 30 from individuals who had sustained a mTBI and healthy control participants. Data from individuals who sustained a mTBI and underwent MRI and were classified as having PPCS at follow-up are identified as red squares; ** *p* < 0.01; (**C**) Scatterplot depicting the partial correlation between the RBANS^®^ Update Visuospatial/Constructional Index Scores at ED Presentation and fractional anisotropy values in the left IFOF threshold of 30 for individuals who had sustained a mTBI and underwent MRI procedures. Data from individuals who underwent MRI procedures and were classified as having PPCS at follow-up are identified as red squares.

Results of the TBSS analyses revealed no statistically significant differences between groups on both the FA (*t*-statistic corrected *p* = 0.683) or MD skeleton (*t*-statistic corrected *p* = 0.601). Similarly, ROI analyses revealed no statistically significant differences between mTBI and healthy control groups for all regions examined in terms of MD values extracted, however, a significant between group difference was found in the FA of the left inferior fronto-occipital fasciculus (IFOF; Figure 6A) (*t* (18.06) = −3.01, *p* = 0.008; Figure 6B). Given that previous reports have indicated that this region is implicated in visuo-spatial constructional ability [102,103,104,105], a partial correlation was conducted between RBANS^®^ Update Visuospatial/Constructional Index Scores, as measured at patient presentation to the ED, and FA values extracted from this region for the mTBI group. Insufficient neuropsychological data was available for healthy controls therefore precluding investigation for this group. Results of the partial correlation indicated a statistically significant correlation between RBANS^®^ Update Visuospatial/Constructional Index Score and extracted FA values from the left IFOF (*r* = 0.63, *p* = 0.038) (Figure 6C) amongst individuals who sustained mTBI. 

## 4. Discussion

The first aim of this pilot study was to univariately examine demographic, neuropsychological, blood-based biomarker and MRI-DTI outcomes for their potential to predict PPCS. In this context, neuropsychological measures and demographic variables appear to show promise. The blood-based biomarkers assessed did not indicate predictive potential and the number of participants assessed for MRI precluded statistical analyses for the intended purpose. Given the small sample size and limited power to detect associations in this pilot study, statistical significance was not the only indicator of potential utility of a variable considered. Failure to detect a significant association is not proof of no association, and this is particularly relevant when power is low. Therefore, variables with substantial estimated effect sizes have been highlighted as warranting further investigation without intention to imply a confirmed association.

The overall incidence of PPCS in this study was consistent with the literature. Of the demographic variables examined, none were identified as statistically significant predictors of PPCS. However, prior history of psychological disorder was estimated to have a positive and substantial OR, which is consistent with findings that have previously been reported with the literature [16,20,37,38]. The magnitude of the estimated ORs for previous history of mTBI and general comorbidities are also thought to be clinically meaningful and should not be dismissed as potential predictors on the basis of *p*-values derived from this pilot. Acknowledging that there was a tendency for relatively less cognitively impaired individuals to return for follow-up in the present study cohort, examination of neuropsychological data identified three components of the RBANS^®^ Update test battery to be predictive of PPCS. These results suggest that this brief neuropsychological screening tool, which can be easily administered bedside in an ED setting by a suitably trained individual, may add significant value to a multi-modal suite of measures that aim to predict PPCS amongst patients who present to hospitals for mTBI. To the authors’ knowledge, the present study was the first of its kind to use the RBANS^®^ Update for predicting PPCS following mTBI. As such, further investigation is warranted in order to validate the use of the RBANS^®^ Update Total Score, Immediate Memory and Attention indices, and potentially the Delayed Memory index, as robust predictors of PPCS. Similarly, the results of the present study suggest that an individual’s performance on the TMT B may also be relevant for the prediction of PPCS and warrants further investigation, particularly in conjunction with assessments of oculomotor function. This is consistent with a previous study by Heitger and colleagues (2007) [106] which identified TMT B performance as a potentially clinically meaningful predictor of PPCS.

The second aim of the present study was to contribute data for blood-based biomarker and MRI-DTI outcomes. None of the three blood-based biomarkers examined were found to be statistically significant predictors of PPCS. However, mean concentration of GFAP at ED presentation differed between mTBI and healthy controls. This finding is consistent with recent reports that suggest that GFAP may be useful for detecting the occurrence of traumatic brain injuries, including mTBI [107,108,109,110,111]. Given that each biomarker is associated with its’ own release kinetic profile, it may be that variation in the time between injury and acquisition of blood samples resulted in the lack of statistically significant differences for the NFL and NSE biomarkers. GFAP is believed to have a relatively stable release kinetic profile, with peak levels occurring approximately 20 h post injury in cases of mild to moderate traumatic brain injury [108] and 1–2 days following incidents of severe traumatic brain injury [111,112,113,114]. In contrast to this, peak levels of NSE appear to be reached 6–12 h post traumatic brain injury [115], while levels of NFL have been observed to peak 144 h following sports-related mTBI [58]. The temporal stability of blood-based biomarkers is increasingly being recognised as a potential limitation to their routine clinical use. Thus, non-protein based biomarkers such as microRNAs may be better suited for the prediction of outcome following mTBI [116,117,118,119].

Given the exploratory nature of the present study and the fact that PPCS affects a relatively small, albeit significant proportion of individuals who sustain a mTBI, an insufficient number of individuals underwent MRI procedures to determine the prognostic utility of the MRI analyses conducted. However, the results obtained are in line with those previously reported, which suggest that the inferior fronto-occipital fasciculus may be particularly vulnerable to the diffuse axonal injury that is present in mTBI [120]. Contrary to previous studies which have observed FA to be decreased in this region [121], our results indicate that relative to healthy controls, FA was, on average, higher amongst participants with mTBI. This increase in FA may be accounted for by the time elapsed between injury and the time at which study participants were imaged. Furthermore, it is also worth noting that in the present study sample, the FA values of the two participants that were classified as having PPCS did not appear to deviate from the other mTBI participants that experienced typical recovery. Additional larger scale studies are required to better establish whether differences in FA can be observed amongst individuals that do and do not recover typically following mTBI, and whether FA may also function as a prognostic biomarker.

Follow-up analyses of neuroimaging data also revealed a statistically significant positive correlation between performance on the RBANS^®^ Update Visuospatial/Constructional index at ED presentation and FA value amongst individuals who had sustained a mTBI. This finding is consistent with previous reports that suggest that the IFOF is implicated in visuospatial functioning. Taking into consideration that mTBI can result in compromised oculomotor function [122], which can also persist in cases of PPCS [123], future studies investigating the association between performance on neuropsychological tests and/or tests of oculomotor function with aberrant MRI-DTI findings, as well as whether microstructural damage within corresponding brain areas may be predictive of PPCS, are warranted. Given the continuous advancement in neuroimaging sequences and analysis techniques, the potential remains for MRI to elucidate biological changes occurring within the brain that result in PPCS.

Having the ability to identify individuals at risk of PPCS has important implications for both clinical and research practice. At the primary healthcare level, it would help clinicians customise treatment plans so that they best meet the unique needs of each individual patient [124] and, most importantly, facilitate the triage of patients to treatment interventions in a more timely manner. Considering that early intervention is anticipated to be integral to optimising patient outcome, and given that PPCS is associated with high utilisation of healthcare services [19,31,43,125,126,127,128], being able to identify at-risk patients and directing them to treatment sooner may assist in reducing the overall burden on the healthcare system that is attributed to PPCS. Furthermore, knowing which individuals may go on to develop PPCS following mTBI would also help clinicians to better manage patient expectations, as it would allow them to provide more accurate advice about the anticipated trajectory of recovery and the academic, occupational, and/or leisure activity accommodations that may be necessary to assist in the process [129]. Prognostic models can also be used to bridge the gap between clinical and research settings. For example, clinicians could use them to direct at-risk patients towards clinical trials of novel therapies that may ameliorate, or even prevent, the development of PPCS and chronic neurodegenerative diseases, such as Alzheimer’s disease [22,23,24] and Chronic Traumatic Encephalopathy [25,27,28,29], that are variably associated with mTBI. Within the context of randomised controlled trials, prognostic models can also be used to increase statistical power through risk stratification and covariate adjustment [31,130,131].

The present study found that recovery status at one-month following mTBI could be predicted by patients’ performance on selected neuropsychological outcome measures when assessed at presentation to the ED. As such, neuropsychological performance may add value to multi-modal prognostic models of PPCS developed in the future. However, PPCS is a multifaceted condition and as performance on neuropsychological tests can also be influenced by extraneous factors, further investigation is needed to examine the utility of additional blood-based and neuroimaging biomarkers in the prediction of PPCS at the individual level.

## Figures and Tables

**Table 1:** Characteristics of participants included in the study and those lost to follow-up.

	Participants Lost to Follow-Up (*n* = 21)		Participants Presenting at Follow-Up (*n* = 39)			
Demographic and Pre-Injury Characteristics	*n*	Missing (*n*)	*n*	Missing (*n*)	*p*	Test
Age: M ± SD	30.62 (7.95)	-	28 (8.89)	-	0.264	*t*-test
Sex: Female (%)	9 (42.86)	-	16 (41.03)	-	0.883	χ^2^
Education (years; M, (SD))	12.68 (2.12)	5	13.74 (1.83)	5	0.089	*t*-test
History of previous mTBI: Yes (%)	12 (57.14)	-	18 (46.15)	-	0.417	χ^2^
History of any psychological disorder: Yes (%)	9 (42.86)	-	11 (28.95)	1	0.274	χ^2^
History of neurological disorder: Yes (%)	1 (4.76)	-	6 (15.39)	-	0.222	χ^2^
History of headaches/migraines: Yes (%)	0 (0)	-	3 (7.69)	-	0.192	χ^2^
General co-morbidities: Yes (%)	7 (33.33)	-	12 (30.77)	-	0.839	χ^2^
Currently on medication: Yes (%)	6 (28.57)	-	11 (28.21)	-	0.976	χ^2^
**Injury Characteristics**	***n***	**Missing (*n*)**	***n***	**Missing (*n*)**		
Loss of Consciousness: Yes (%)	9 (60)	6	22 (66.67)	6	0.433	χ^2^
Δ time between injury and ED assessment (hours; M (SD))	10.48 (6.57)	3	8.75 (7.20)	2	0.409	*t*-test
**Performance on Neuropsychological Measures at Presentation to ED**	**Mean (SD)**	**Missing (*n*)**	**Mean (SD)**	**Missing (*n*)**		
RMPCQ	22.57 (14.68)	-	18.38 (10.82)	-	0.213	*t*-test
RBANS^®^ Update Total Score	80.78 (13.46)	3	92.16 (13.13)	2	0.004	*t*-test
RBANS^®^ Update Immediate Memory	73 (14.57)	3	88.28 (15.37)	0	0.001	*t*-test
RBANS^®^ Update Visual Constructional	94.35 (17.66)	1	98.45 (17.40)	1	0.400	*t*-test
RBANS^®^ Update Attention	80.06 (18.55)	1	89.46 (16.65)	2	0.069	*t*-test
RBANS^®^ Update Language	94.60 (10.56)	1	99.59 (15.07)	0	0.192	*t*-test
RBANS^®^ Update Delayed Memory	86.47 (13.29)	1	90.19 (10.89)	2	0.282	*t*-test
TMT B Completion time (sec)	85.91 (47.37)	4	54.87 (14.77)	1	0.017	*t*-test
DASS-21 Total Score	17.05 (16.01)	-	11.58 (8.73)	-	0.159	*t*-test
DASS-21 Depression Subscale	5.62 (5.97)	-	3.31 (3.89)	-	0.120	*t*-test
DASS-21 Anxiety Subscale	5.05 (5.34)	-	3.50 (3.00)	-	0.231	*t*-test
DASS-21 Stress Subscale	6.24 (5.70)	-	4.73 (3.31)	-	0.276	*t*-test
RMT	13.38 (2.16)	5	14.13 (1.48)	7	0.163	*t*-test

Note: M: mean; SD: standard deviation; RMPCQ: Rivermead Post-Concussion Symptoms Questionnaire; RBANS^®^ Update: Repeatable Battery for the Assessment of Neuropsychological Status, Update; TMT B: Trails Making Test version B; DASS-21: Depression, Anxiety and Stress Scales- 21 item version; RMT: Rey Malingering Test.

**Table 2:** Demographic variables, injury-related characteristics, and performance on neuropsychological outcome measures of participants who recovered typically and those who developed PPCS, with exact logistic regression odds ratios for PPCS.

	mTBI Typical Recovery (*n* = 33)		PPCS (*n* = 3)				
Demographic Variable	*n*	Missing (*n*)	*n*	Missing (*n*)	OR	95% CI	*p*
Age (years): M (SD)	28.64 (9.09)	-	21 (2.65)	-	0.80	0.52–1.03	0.122
Range	18–49		18–23				
Sex: Female (%)	14 (42.40)	-	1 (33.33)	-	0.69	0.01–14.42	1.000
Years of education: M (SD)	13.89 (1.99)	5	12.33 (1.53)	-	0.85	0.14–7.85	1.000
Range	10–17		11–14				
<12 years education (%)	9 (27.30)	5	2 (66.67)	-	0.25	0–5.37	0.563
History of previous mTBI: Yes (%)	16 (48.50)	-	3 (100)	-	3.76 *	0.38–†	0.271
Number of previous mTBI	1 previous mTBI: *n* = 10		1 previous mTBI: *n* = 2		1.33	0.58–2.69	0.444
	≥2 previous mTBI: *n* = 6		≥2 previous mTBI: *n* = 1				
History of any psychological disorder: Yes (%)	9 (27.30)	1	2 (66.67)	-	4.84	0.23–314.29	0.454
History of neurological disorder: Yes (%)	5 (15.20)	-	0 (0)	-	1.58	0–16.86	1.000
History of headaches/migraines: Yes (%)	3 (9.10)	-	0 (0)	-	2.90	0–34.44	1.000
General co-morbidities: Yes (%)	9 (27.30)	-	2 (66.67)	-	5.05	0.24–327.39	0.431
Currently on medication: Yes (%)	9 (27.30)	-	1 (33.33)	-	1.32	0.02–28.44	1.000
Smoker: Yes (%)	7 (21.20)	1	1 (33.33)	-	1.75	0.03–38.57	1.000
>10 cigarettes/day	5 (15.20)	1	0 (0)	-	1.53	0–16.29	1.000
Exercise each week: Yes (%)	31 (93.90)	-	2 (66.67)	-	0.14	0.01–11.39	0.472
Number of hours exercised/week: M (SD)	14.67 (14.73)	-	22.67 (20.53)	-	1.03	0.96–1.11	0.398
Alcohol consumer: Yes (%)	23 (69.70)	3	2 (66.67)	-	0.62	0.03–40.99	1.000
Number of standard drinks consumed per week: M (SD)	4.38 (4.95)	4	4.67 (5.03)	-	1.01	0.76–1.27	0.870
**Injury-related Characteristics**		**Missing (*n*)**		**Missing (*n*)**	**OR**	**95% CI**	***p***
Loss of consciousness: Yes (%)	20 (60.60)	3	1 (50)	1	0.51	0.01–43.14	1.000
Δ time between injury and ED assessment (hours; M (SD))	10.84 (10.66)	1	11.08 (10.47)	-	1.00	0.88–1.11	0.810
**Performance on Neuropsychological Outcomes at ED Presentation**	**Mean (SD)**	**Missing (*n*)**	**Mean (SD)**	**Missing (*n*)**	**OR**	**95% CI**	***p***
RBANS^®^ Update Total Score	94.12 (12.38)	-	73.00 (9.84)	-	0.81	0.61–0.095	0.004
RBANS^®^ Update Immediate Memory	91.76 (13.57)	-	63.67 (12.22)	-	0.79	0.55–0.94	0.001
RBANS^®^ Update Visual Constructional	100.61 (16.85)	-	93.67 (8.51)	-	0.97	0.90–1.05	0.498
RBANS^®^ Update Language	102.67 (11.82)	-	92.67 (8.15)	-	0.92	0.81–1.03	0.174
RBANS^®^ Update Attention	91.52 (15.58)	-	65.33 (12.86)	-	0.86	0.71–0.97	0.007
RBANS^®^ Update Delayed Memory	92.45 (12.24)	-	79.00 (3.46)	-	0.90	0.79–1.01	0.071
TMT B Completion Time (sec)	56.83 (19.34)	-	87.70 (20.54)	-	1.06	1.00–1.12	0.032
DASS-21 Total Score	11.94 (10.36)	-	12.67 (6.35)	-	1.01	0.88–1.12	0.838
DASS-21 Depression	3.36 (4.05)	-	3.00 (3.61)	-	0.98	0.65–1.31	1.000
DASS-21 Anxiety	3.48 (3.81)	-	4.67 (1.16)	-	1.08	0.77–1.42	0.562
DASS-21 Stress	5.09 (3.96)	-	5.00 (2.00)	-	0.99	0.69–1.35	1.000
RMT	14.39 (1.37)	5	12.50 (0.71)	1	0.52	0.17–1.37	0.202

Note: M: mean; SD: standard deviation; * median unbounded estimate; † 95% CI upper limit not definable; RMPCQ: Rivermead Post-Concussion Symptoms Questionnaire; RBANS^®^ Update: Repeatable Battery for the Assessment of Neuropsychological Status, Update; TMT B: Trails Making Test version B; DASS-21: Depression, Anxiety and Stress Scales- 21 item version; RMT: Rey Malingering Test; ORs have been calculated per one unit increase for all continuous variables.

**Table 3:** Exact logistic regression odds ratios for PPCS for blood-based biomarkers.

	mTBI (*n* = 33)		PPCS (*n* = 3)				
Blood-Based Biomarker	*n*	Mean (SD)	*n*	Mean (SD)	OR	95% CI	*p*
GFAP (pg/mL)	27	482.12 (553.95)	3	231.00 (139.31)	0.998	0.992–1.002	0.540
GFAP (50 pg/mL)	27	9.64 (11.08)	3	4.62 (2.79)	0.905	0.669–1.105	0.540
NFL (pg/mL)	32	5.89 (2.28)	3	6.33 (4.38)	1.075	0.650–1.688	0.706
NFL (50 pg/mL)	32	0.12 (0.04)	3	0.13 (0.09)	37.19	4.42 × 10^−10^–2.34 × 10^11^	0.706
NSE (pg/mL)	32	5950.88 (4476.00)	3	7939.33 (4921.24)	1.00008	0.9998–1.0003	0.417
NSE (50 pg/mL)	32	119.02 (89.52)	3	158.79 (98.43)	1.004	0.9900–1.015	0.417

Note: Odds ratios have been calculated per one unit increase for all blood-based biomarkers examined. Means and standard deviations are provided for descriptive purposes only.

**Table 4:** Conditional logistic regression odds ratios for mTBI for blood-based biomarkers.

	mTBI (Total *n* = 36)		Healthy Controls (Total *n* = 36)				
Blood-Based Biomarker	*n*	Mean (SD)	*n*	Mean (SD)	OR	95% CI	*p*
GFAP (pg/mL)	30	457.01 (531.35)	30	96.68 (35.43)	1.028	1.001–1.056	0.042
GFAP (50 pg/mL)	30	9.14 (10.63)	30	1.94 (0.71)	3.978	1.051–15.247	0.042
NFL (pg/mL)	36	5.92 (2.42)	36	5.41 (1.93)	1.125	0.90–1.41	0.310
NFL (50 pg/mL)	36	0.12 (0.05)	36	0.11 (0.04)	361.099	0.005–2.89 × 10^7^	0.310
NSE (pg/mL)	35	6121.31 (4473.30)	35	4675.26 (2179.96)	1.0001	1.0000–1.0002	0.144
NSE (50 pg/mL)	35	122.43 (89.47)	35	93.51 (43.60)	1.005	1–1.01	0.144

Note: Odds ratios have been calculated per one unit increase for all blood-based biomarkers examined. Means and standard deviations are provided for descriptive purposes only.
